# Co-occurrence network analysis reveals novel associations between the neonatal airway microbiome and bronchopulmonary dysplasia risk: an observational, population-based study

**DOI:** 10.1128/msphere.00857-25

**Published:** 2026-02-03

**Authors:** Liang Gao, Yingying Qiu, Xinzhu Lin, Yulin Zhou, Yvcong Lin, Kunyao Hong, Lian Wang, Wei Shen, Qian Zhang

**Affiliations:** 1 Department of Neonatology, Women and Children’s Hospital, School of Medicine, Xiamen University 205357, Xiamen, Fujian, China; 2 Xiamen Key Laboratory of Perinatal-neonatal Infection, Xiamen, China; 3 Department of pediatrics, Women and Children’s Hospital, School of Medicine, Xiamen University 205357, Xiamen, Fujian, China; 4 School of Public Health, Xiamen University 205357, Xiamen, Fujian, China; 5 College of the Environment and Ecology, Xiamen University 598522, Xiamen, Fujian, China; 6 Key laboratory of Ministry of Education for Coastal and Wetland Ecosystems, Xiamen University, Xiamen, Fujian, China; 7 Fujian Provincial Key Laboratory for Coastal Ecology and Enviromental Stusdies, Xiamen University, Xiamen, Fujian, China; University of Michigan-Ann Arbor, Ann Arbor, Michigan, USA

**Keywords:** bronchopulmonary dysplasia, very preterm infant, lower airway, microbiota

## Abstract

To evaluate the association between respiratory tract microorganisms at birth and the subsequent development and severity of bronchopulmonary dysplasia (BPD) in preterm infants. This prospective cohort study enrolled 98 preterm infants (gestational age < 32 weeks, birth weight < 2,000 g). Tracheal aspirate samples were collected through endotracheal intubation within 2 h after birth. Using 16S rRNA sequencing, we characterized the airway microbiome and performed co-occurrence network analysis with compositionally robust methods. Among 98 preterm infants analyzed, the incidence of BPD was 68.4%, comprising 31 grade I, 20 grade II, and 16 grade III cases. Airway microbiota in infants with BPD exhibited distinct severity-stage patterns: *Escherichia-Shigella* and *Streptococcus* were significantly enriched in grade I, while *Chryseobacterium* increased markedly in grade III, accompanied by a significant reduction in *Streptococcus*. Microbial co-occurrence network analysis yielded three key insights. (i) Network complexity declined sharply with BPD severity, being sparsest in grade III. (ii) Distinct keystone taxa were identified across different groups: *Acinetobacter* and *Fusobacterium* in the non-BPD group; *Brevundimonas* and *Fusobacterium* in grade I; *Fusobacterium* and *Acinetobacter* at grade II and grade III. (iii) In a multivariable model adjusted for key clinical confounders, a higher microbial network density at birth was independently associated with a substantially reduced risk (OR = 0.12, *P* < 0.05). The ecological architecture of the neonatal airway microbiome at birth, defined by network complexity and keystone taxa, is associated with BPD severity. This highlights microbial network stability as a novel factor and ecological interactions as a target for future research.

**IMPORTANCE:**

Bronchopulmonary dysplasia (BPD) remains the most common chronic lung disease in preterm infants. While its pathogenesis is incompletely understood, the role of the early respiratory microbe is increasingly recognized. Previous studies have largely focused on individual pathogenic taxa, overlooking the complex ecological interactions within microbial communities. Our analysis reveals that the architecture of microbial co-occurrence networks in the neonatal airway varies significantly with BPD severity. Notably, network complexity decreased markedly as BPD severity increased. We identified specific keystone taxa uniquely associated with disease outcomes, suggesting that microbial ecosystem stability rather than individual species may be a critical factor in BPD pathogenesis. These findings shift the focus from single microbes to the stability of the microbial ecosystem as a novel risk factor for severe BPD, offering new avenues for risk stratification and early intervention.

## INTRODUCTION

Bronchopulmonary dysplasia (BPD) is a chronic lung disease of newborns and the most common complication of preterm infants. Despite advances in neonatal care, the incidence of BPD remains high, affecting approximately 56.2% of infants born at gestational age (GA) less than 28 weeks and 29.2% of those born at GA less than 32 weeks (1). BPD has adverse effects on the cardiopulmonary function and neurodevelopment of preterm infants (2, 3), aggravating the public health burden. Given the severity of adverse outcomes associated with BPD, it is important to identify factors influencing the development of BPD and explore potential strategies to improve neonatal care and the long-term health status of preterm infants. BPD pathogenesis involves genetic predisposition, intrauterine and postnatal inflammation, infection, and perinatal factors. A systematic review that included six studies suggested that dysbiosis of the airway microbiome may be associated with the progression and severity of BPD (4). The detection rate of Gram-negative bacteria in tracheobronchial aspirate fluid was significantly higher in preterm infants with severe BPD than in those without severe BPD (5). In addition, Gram-negative bacterial colonization in the airway was associated with an increased risk of death or supplemental oxygen at discharge in infants with severe BPD and increased the risk of long-term adverse outcomes in patients with BPD (6).

Microorganisms in microbial communities interact to promote community stability (7). However, how microorganisms interact to influence the occurrence of BPD remains largely unknown. Co-occurrence network analysis, which uses graph theory to illustrate potential interactions between communities, is a powerful tool to study the complex interactions between different microbial groups within communities (8). In this study, we characterized the lower respiratory tract (LRT) microbiome at birth in preterm infants and applied co-occurrence network analysis to explore its relationship with BPD severity. We hypothesized that specific microbial colonization patterns, particularly involving keystone taxa, are associated with BPD occurrence and severity.

## MATERIALS AND METHODS

### Study design and participants

This prospective cohort study was conducted at Xiamen Women and Children’s Hospital (Xiamen, Fujian, China) between February 2022 and March 2024.

### Inclusion and exclusion criteria

Infants were eligible for inclusion if they met all the following criteria: (i) GA between 24+0/7 and 31+6/7 weeks with a birth weight of 400–2,000 g; (ii) requirement for endotracheal intubation within 2 h after birth; (iii) single respiratory secretion specimen volume ≥0.5 mL; (iv) obtained informed consent and a signed written authorization form from the infant’s legal guardian. Exclusion criteria included (i) severe congenital respiratory structural abnormalities, including congenital heart disease, diaphragmatic hernia, pulmonary sequestration, or pulmonary cystic adenoma; (ii) death from non-respiratory diseases with corrected GA below 36 weeks; (iii) systemic antimicrobial therapy administered before sample collection; (iv) intratracheal administration before sample collection; (v) initiation of enteral feeding before sample collection; (vi) meconium-stained amniotic fluid or bloody amniotic fluid. A follow-up was conducted until discharge or until the corrected GA reached 36 to 40 weeks.

### Sample size consideration

All participants in this study received tracheal intubation at NICUs within 2 h of birth, following standardized criteria for receiving tracheal intubation for the comparability among all subjects (9). Based on the 2021 CHNN report, which indicated a BPD incidence of approximately 29.2% among very preterm infants in mainland China (1), this exploratory study planned to enroll 100 cases to ensure a sufficiently large cohort for initial microbial ecology analysis.

### Specimen collection and processing

**Fig 1:**
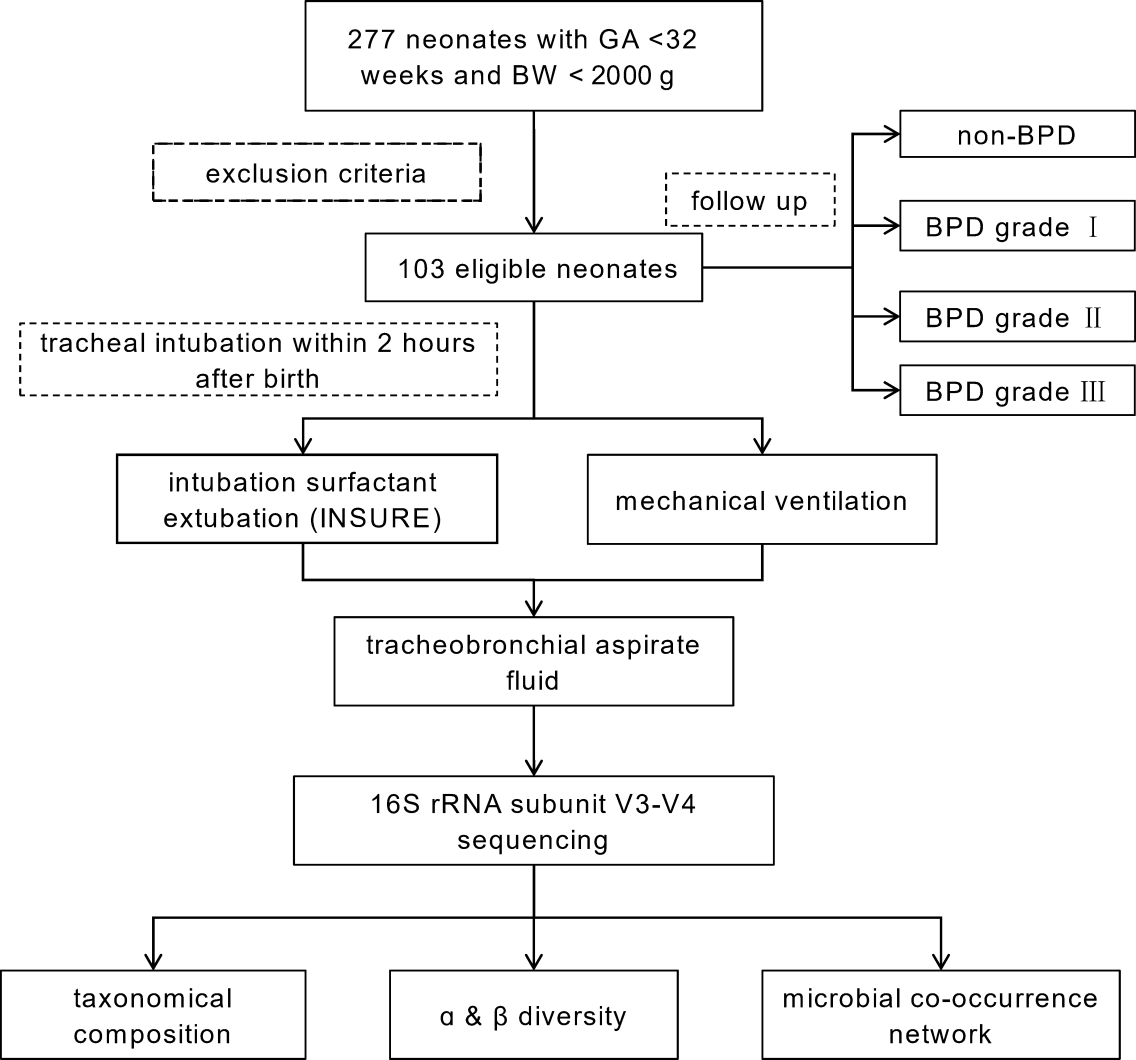
Technical methodology flow diagram.

LRT secretion specimens were collected under sterile conditions during endotracheal intubation using closed suction within 2 h of birth. A minimum volume of 0.5 mL per sample was required to ensure sufficient microbial biomass for downstream analysis (Fig. 1). All collected samples were immediately stored at −80°C until DNA extraction. Detailed protocols for DNA extraction, sequencing, and bioinformatic processing are provided in the supplemental methods.

### Clinical outcomes

The primary outcome was the severity of BPD in participants. This study followed the diagnostic and severity grading criteria for BPD established by the Eunice Kennedy Shriver National Institute of Child Health and Human Development, United States (10). The standard defines BPD as persistent parenchymal lung disease (diagnosed by radiographic examination) that requires any form of respiratory support (including invasive or non-invasive ventilation or supplemental oxygen) at 36 weeks of gestation. BPD is classified into grades I, II, and III based on different fractions of inspired oxygen (FiO_2_) ranges. It also includes early deaths owing to persistent parenchymal lung disease and respiratory failure that cannot be attributed to other neonatal morbidity (Table S7).

### Extraction and sequencing

Genomic DNA was extracted from 103 clinical samples, along with one kit control, using the FastDNA Spin Kit for Soil (MP Biomedicals, USA) following the manufacturer's protocol. Of the 103 samples, 98 yielded high-quality genomic DNA, which was subsequently verified by agarose gel electrophoresis. To monitor potential contamination, sterile water was included as the negative control during DNA extraction and PCR amplification. The absence of detectable amplification in these controls, confirmed by agarose gel electrophoresis, supports the specificity of the microbial profiles obtained from clinical samples. The V3 and V4 region (338F: 5′-ACTCCTACGGGAGGCAGCAG-3′, 806R: 5′-GGACTACHVGGGTWTCTAAT-3′) of the 16S ribosomal RNA gene was sequenced on an Illumina MiSeq platform (San Diego, CA, USA). Briefly, PCR was conducted in triplicate under the following conditions: 95°C for 3 min, followed by 30 cycles at 95°C for 30 s, 55°C for 30 s, and 72°C for 45 s and a final extension at 72°C for 10 min. All sequencing libraries were prepared uniformly using the NEXTFLEX Rapid DNA-Seq Kit (Bioo Scientific), and paired-end sequencing was performed on a single Illumina MiSeq PE300 platform using the same reagent kit type (MiSeq Reagent Kit v3). This standardized workflow further reduces technical variability.

### Bioinformatics processing

Further preprocessing of sequence data was performed on the QIIME2 pipeline. Quality control and filtering of paired-end reads were conducted based on sequencing quality, and splicing was carried out according to the overlap relationship between paired-end reads to obtain optimized data after quality control splicing. Then, sequence denoising methods (DADA2) were used to process the optimized data, obtaining amplicon sequence variant (ASV) representative sequences and abundance information. To mitigate the impact of potential contaminants and sequencing artifacts, we applied a conservative filtering approach. All features (ASVs) with a total frequency of one (singletons) were removed, as were sequences taxonomically classified as mitochondrial or chloroplast. The taxonomic assignments were updated subsequent to this filtering step. ASVs were clustered against the Silva reference database (ver. 138.2), and their taxonomic identities were assigned using the RDP classifier (version 2.11; https://github.com/rdpstaff/classifier) with the naive Bayes algorithm, which was trained on the SILVA v138.2 reference sequences. Specifically, we extracted primer-specific reference sequences from the full-length 16S sequences in the Silva database. We then compared the trained classifier with the representative sequences of bacteria identified by DADA2 analysis to obtain bacterial taxonomic information.

### Statistical analysis

We assessed associations between the severity of BPD and key features of the airway bacterial microbiome: community composition, alpha diversity, beta diversity, and the bacterial co-occurrence network. Alpha diversity indices, including Chao1 species richness estimate, Shannon index, Simpson index, and Good’s coverage index, were calculated by using Mothur software (ver. 1.48.0) (11). Beta diversity among various BPD grades was evaluated by analysis of similarity (ANOSIM) using Bray-Curtis dissimilarity matrices in Mothur. ANOSIM *P* values were adjusted by Bonferroni correction for multiple comparisons. Ordination of Bray-Curtis distance was performed by using constrained principal coordinate analysis (CPCoA) through the capscale function implemented using the vegan package (ver. 2.6.0) in R Statistical Software (ver. 4.4.2) (12). Potential clinical confounding factors were statistically controlled for during the analysis. Specifically, the PERMDISP test was first used to assess the homogeneity of dispersion among groups, ensuring the validity of subsequent PERMANOVA results. Missing data were imputed using a random forest-based imputation method, implemented via the missForest package (ver. 1.5) in R (13). This non-parametric method uses an iterative approach to train random forest models on observed data to predict missing values, handling both continuous and categorical variables and accounting for potential nonlinear relationships. Statistical analyses were performed using SPSS (ver. 26.0). Continuous variables were assessed using the Kruskal-Wallis H-test after a normality test by the Shapiro-Wilk test and a variance homogeneity test by Levene’s test. Categorical variables were compared by the Pearson chi-square test or Fisher’s exact test. Genus-level differential abundance was assessed using ANCOM-BC (Analysis of Compositions of Microbiomes with Bias Correction), enabling bias-corrected effect size estimates and confidence intervals for each genus across the BPD groups (14). A *P* value less than 0.05 was considered statistically significant.

Only features with a sum of relative abundance greater than 0.01% and prevalence in 5% of samples (5/98) were retained in the network analysis. These filters were applied to minimize the influence of potential sequencing artifacts and low-abundance contaminants and to enhance the numerical stability of the subsequent compositional correlation analysis. To mitigate biases in co-occurrence network inference arising from unequal group sizes and to ensure computational stability, we normalized the sample size across groups. Each group was down-sampled randomly to *N* = 16, matching the size of the smallest group. This approach ensures that network estimates are not disproportionately influenced by larger groups. The CONSORT figure is supplied as Fig. S1. To account for the compositional nature of microbiome data, microbial co-occurrence networks were constructed using the Sparse Correlations for Compositional Data (SparCC) algorithm (v0.1.0), a compositionally robust correlation method implemented in Python (15). SparCC correlations were calculated from normalized relative abundance data with 20 iterations to ensure convergence. Correlation significance and edge stability were assessed through 100 bootstrap resamplings, and only significant edges (*P* < 0.05) were retained for subsequent network construction. Network construction employed a threshold of |*r*| >0.3 to define significant microbial associations. This threshold was selected based on established practices for SparCC in microbial ecology and its suitability for capturing strong, biologically plausible interactions in compositional data (16). The robustness of the results was verified through multi-threshold sensitivity analysis (using thresholds of |*r*| > 0.2 and |*r*| > 0.4). To evaluate the impact of methodological choice, we systematically compared networks inferred by SparCC with those generated using Spearman’s correlation on relative abundances, with similarity assessed using the Jaccard index. Network topological properties were analyzed using igraph (17). In networks, ASVs represent nodes, the size of the node indicates relative abundance, and correlations between ASVs are displayed as edges. The parameters used in this study included number of nodes (the number of ASVs), number of edges (the number of connections among all of the nodes), average degree (the average number of connections per node), average path length (average number of steps along the shortest paths between nodes), diameter (the longest shortest path between any two nodes), edge density (the ratio of actual edges to the maximum possible edges), clustering coefficient (the extent of nodes in a network associate with other nodes in the network), modularity, betweenness centralization (the number of times a node acts as a bridge along the shortest path between two other nodes), closeness centralization (the reciprocal of the distance of the nodes to all other nodes) and eigenvector centralization (the degree to which centrality is concentrated among a few dominant nodes). In addition, the degree, betweenness centrality, closeness centrality, and assortativity of a single node in the network are calculated.

Node-level centrality was assessed through degree, betweenness, and closeness centrality. Extensive research in microbial ecology has consistently shown that keystone species within co-occurrence networks tend to display distinct topological patterns, specifically featuring high degree centrality, low betweenness centrality, high closeness centrality, and high transitivity (18, 19). Accordingly, we assigned differential weights to each centrality measure based on their specific ecological interpretations to refine the identification. Taxa in the top 10% of this composite keystone score were designated as keystone taxa.

Based on the identified keystones, we further constructed an ordered logistic regression model to correlate relative abundance of key taxa with BPD severity. 11 potential confounding factors selected based on clinical importance and differential variables were included: gender, GA, BW, delivery mode, antibiotic type, antenatal corticosteroids (ACS), chorioamnionitis, meconium-stained fluid (MSF), extrauterine growth restriction (EUGR), intrauterine distress, the length of hospital stay. We performed a logarithmic transformation and standardized the abundance data to variables with a mean of 0 and a standard deviation of 1, while also applying standardization to numerical variables. Due to sample size limitations and multicollinearity issues, we employed forward stepwise variable selection. Starting with a baseline model containing only ASV, we sequentially added covariates in order of clinical importance. At each step, we checked model convergence and numerical stability, excluded variables causing Hessian matrix singularity, and ultimately retained covariate combinations that maintained model stability.

Visualization of the co-occurrence network was performed by using Gephi (20). We utilized the network dissimilarity coefficient to evaluate the overall network dissimilarity among different severities of BPD (21). We further used NetCoMi to assess parameter differences in network topological features (22). The Jaccard index was used to evaluate the similarity of degree and centrality, ranging from 0 to 1, with higher values indicating greater similarity. For each group, 1,000 random permutations were performed, and the statistical significance threshold was set at *P* <0.05. The ComplexHeatmap package was used to visualize differences in network topology features across groups (23).

The multivariable ordinal logistic regression model was developed using the polr function from the R MASS package, employing a forward stepwise selection approach to assess the association between network topology metrics and the severity of BPD. All continuous network topology metrics, including edge number, diameter, average path length, average nearest neighbor degree, betweenness centralization, density, degree centralization, degree assortativity, and transitivity, were standardized using Z-scores to eliminate scale effects. The model incorporated the following covariates as potential confounders: gender, GA, BW, delivery mode, antibiotic type, ACS, MSF, EUGR, intrauterine distress, and the length of hospital stay.

## RESULTS

Figure 1 briefly presents the methodology and results of this cohort study. Between 26 February 2022 and 04 March 2024, we enrolled 277 preterm infants with GA <32 weeks and BW <2,000 g. One hundred eighty-three were treated with early tracheal intubation after birth. Excluding 3 who accepted resuscitation and administration in the delivery room, 30 had their first tracheal intubation more than 2 h after delivery, 12 received anti-infective drugs before sample collection, and 35 had a single sample volume less than 0.5 mL. After exclusion, 103 samples met the study requirements and were included for subsequent analysis, with 98 (95.15%) successfully sequenced for 16S rRNA amplicon analysis.

### Clinical characteristics

The study population included more males (44 [65.7%] of 67 with BPD and 20 [64.5%] of 31 without BPD) than females (23 [34.3%] with BPD and 11 [35.5%] without BPD), and the GA was 30.57 weeks (SD 1.405) for non-BPD, 29.13 weeks (SD 1.700) for grade I, 29.48 weeks (SD 1.758) grade II and 27.78 weeks (SD 2.425) for grade III. The morbidity of BPD was 68.4% (67/98), with 31 (31.6%) grade I, 20 (20.4%) grade II, and 16 (16.3%) grade III. There were significant differences in the GA, BW, and the mode of delivery among the infants with different severities of BPD (Table S1).

Given the adverse effects of BPD on the cardiopulmonary function and neurodevelopment of preterm infants, we collected information on clinical outcomes for participants. All infants were administered antibiotic monotherapy or combination therapy for pneumonia. Monotherapy antibiotics only include the use of β-lactams, and combination therapy refers to the combination of β-lactams with other antibiotics, like glycopeptides and macrolides. Most participants (91.8%) received pulmonary surfactant treatment for respiratory distress. There were differences in the length of hospital stay (*P* < 0.001) and type of antibiotic therapy (*P* = 0.031) based on treatment type (Table S2). There were also significant differences among infants with different outcomes in the frequencies of hsPDA (*P* = 0.004) and EUGR (*P* = 0.004) (Table S3).

### Microbiome profiles

**Fig 2:**
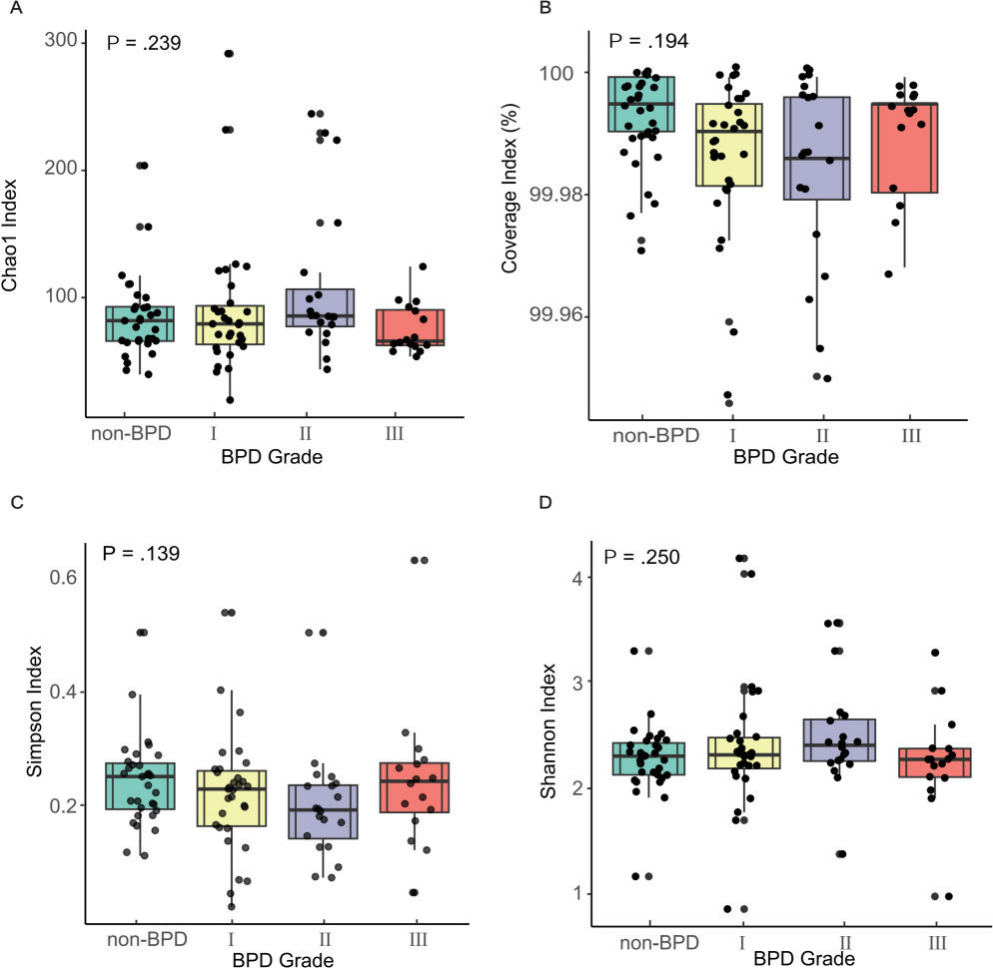
Alpha diversity indices across BPD grades in preterm infants. Diversity of bacterial communities in tracheal aspirate is evaluated by Chao1 species richness estimate (**A**), Good’s coverage index (**B**), Simpson index (**C**), and Shannon index (**D**). The box plot shows the upper and lower quartiles and the mean values.

**Fig 3:**
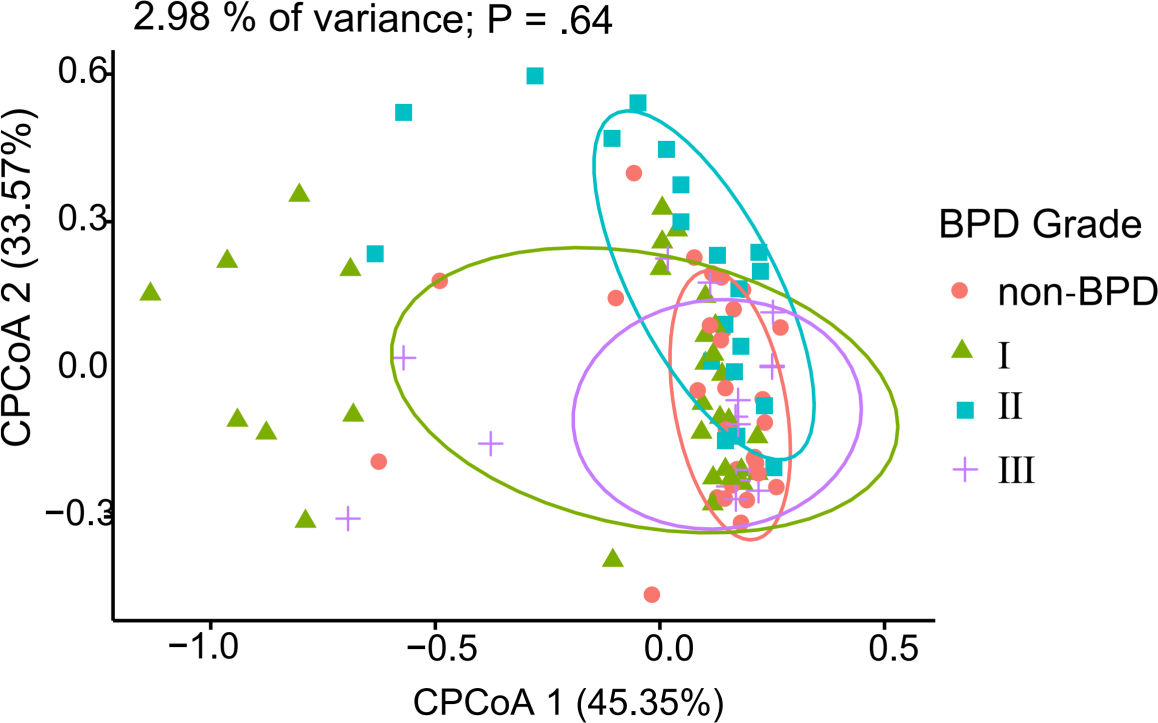
CPCoA of microbial community structure by BPD grades. Each point represents a tracheal aspirate sample. The analysis is based on Bray-Curtis distances. Axes indicate the percentage of explained variance. *P* value is calculated by the PERMANOVA test.

A total of 6,182,749 sequencing reads were obtained from 98 samples for 16S rRNA analyses after quality control filtering and removal of potential human DNA contamination, with reads ranging from 22,026 to 72,630 per sample. In total, 2,894 ASVs were inferred by a *de novo* process, distinguishing sequence variants differing by as little as one nucleotide after a rarefaction depth of 22,000. However, no significant differences in alpha and beta diversity were observed among the samples collected at birth (Fig. 2 and 3). For Chao1 richness, the Kruskal-Wallis test yielded *R*^²^ = 4.22 (*P* = 0.24), indicating minimal between-group differences. Similarly, Shannon diversity showed *R*^²^ = 4.11 (*P* = 0.25), Simpson diversity *R*^²^ = 5.49 (*P* = 0.14), and coverage index *R*^²^ = 4.71 (*P* = 0.19). This indicates that less than 5% of the variance in alpha diversity can be attributed to group differences. The 95% confidence intervals for between-group mean differences all encompass zero, further supporting the absence of meaningful diversity variations among samples collected at birth. The PERMDISP test indicates similar within-group variability (*F* = 0.93, *P* = 0.43), making the PERMANOVA test applicable. PERMANOVA analysis revealed no significant difference in the BPD Group's unique contribution to microbial composition (*F* = 0.89, *P* = 0.64). Further examination of individual covariates showed that antimicrobial type was the primary driver of microbial variation, explaining 19.3% of the variation (*R*^²^ = 0.19).

**Fig 4:**
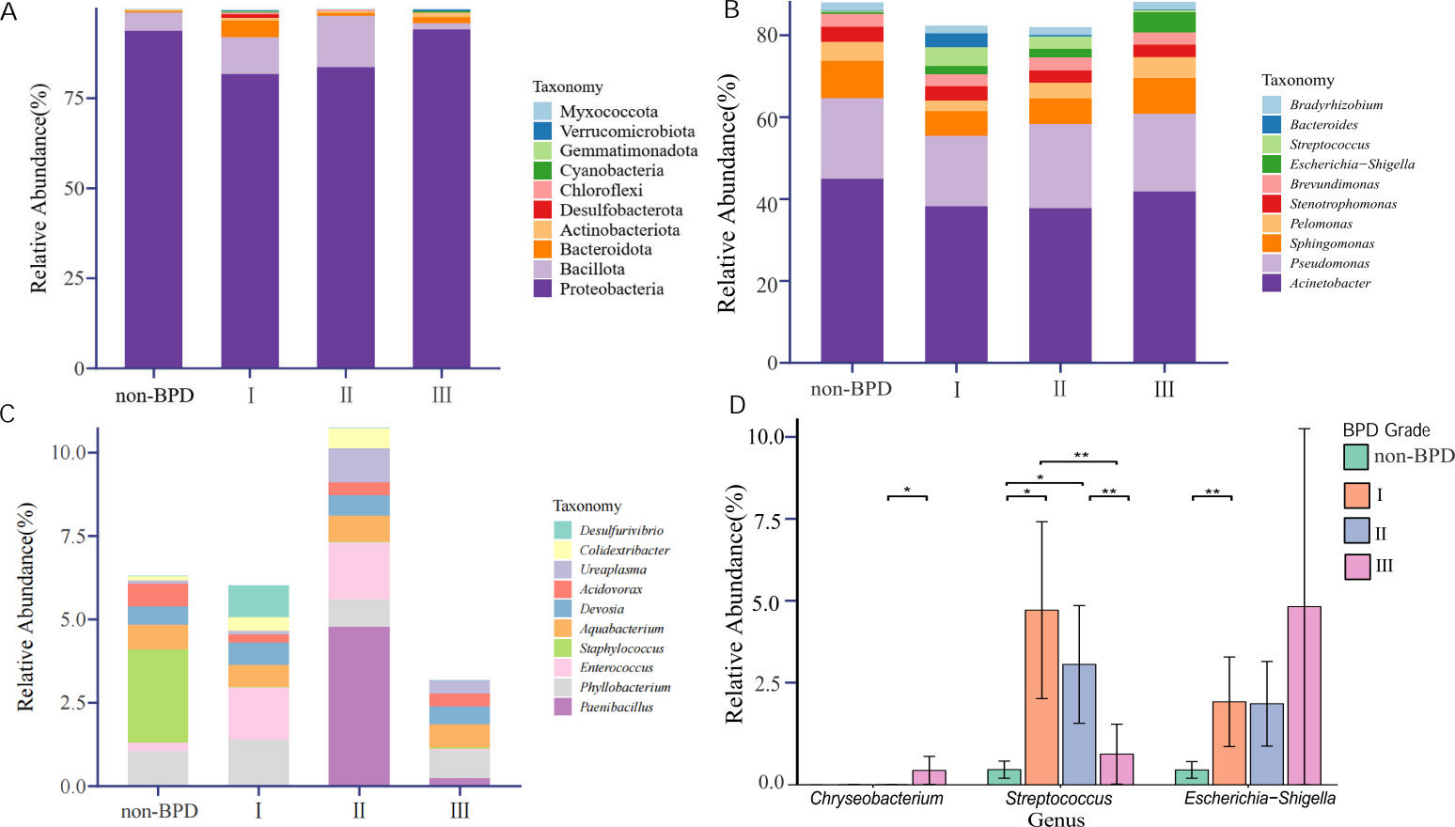
Relative abundance of microbial taxa at phylum and genus levels across BPD grades. The average of major and minor relative abundance of the taxonomic structure at the phylum (**A**) and genus (**B, C**) levels is shown by the stacked bar chart. Genera are split into two panels: (**B**) major and (**C**) minor groups, based on their average abundance. Comparison of significant differences in relative abundance among three genera (**D**). * and ** denote significant differences (*q* < 0.05 and *q* < 0.01, respectively) between specific groups as detailed in Table S5. Data are presented as mean ± SEM.

Among the 33 bacterial phyla identified across all samples, Proteobacteria exhibited the highest relative abundance (mean 87.9%, median 98.7% [IQR 93.6–99.3]), followed by Bacillota (8.2%, 0.4% [0.1–4.1]), Bacteroidota (1.9%, 0.3% [0.2–0.6]), and Actinomycetota (0.7%, 0.2% [0.1–0.3]) (Fig. 4A). No significant differences were observed in the relative abundance of these phyla among the four groups classified by BPD severity. Of the 566 genera detected in all samples, *Acinetobacter* was the most abundant (41.5%, 47.9% [34.9–54.0]), followed by *Pseudomonas* (19.3%, 21.8% [17.1–24.8]), *Sphingomonas* (7.6%, 6.6% [3.5–8.8]), *Pelomonas* (4.0%, 1.6% [1.1–3.9]), and *Stenotrophomonas* (3.5%, 3.4% [2.7–4.0]). As BPD severity increased, *Escherichia-Shigella* tended to increase in relative abundance, while *Bacteroides* showed a decreasing trend (Fig. 4B). *Staphylococcus* was detected exclusively in the non-BPD group, whereas *Enterococcus* showed increased relative abundance in BPD groups, except for grade III (Fig. 4C). Notably, the relative abundances of *Aquabacterium* and *Devosia* remained stable across different BPD severities, showing no statistically significant fluctuations. ANCOM-BC analysis identified three genera with differential abundance across BPD severity groups (Table S5). *Escherichia-Shigella* exhibited a significant abundance increase in the grade I group compared to the non-BPD group (lfc = 2.09, *q* = 0.004). *Streptococcus* was significantly increased in both the grade I (lfc = 1.78, *q* = 0.048) and II (lfc = 2.09, *q* = 0.028) groups, but was significantly decreased in the grade III group versus the grade I (lfc = −2.30, *q* = 0.006) and II (lfc = −2.61, *q* = 0.003) groups. *Chryseobacterium* showed a marked increase in the grade III group versus the grade II (lfc = 1.83, *q* = 0.011) (Fig. 4D).

### Microbial co-occurrence network

We conducted network analyses to explore the microbial co-occurrence patterns in the lower airway microbiome at ASV levels. Given the sample size, the following network comparisons are presented as an exploratory analysis of microbial community structures associated with BPD severity. The sensitivity analysis revealed that the global topology of the co-occurrence networks and the identification of keystone taxa were highly robust to the choice of correlation thresholds in the healthy control group. In contrast, networks in BPD groups showed greater variability, underscoring the ecological impact of the disease. Based on these findings, the moderate threshold (|*r*| > 0.3, FDR < 0.05) was employed for all downstream analyses.

**Fig 5:**
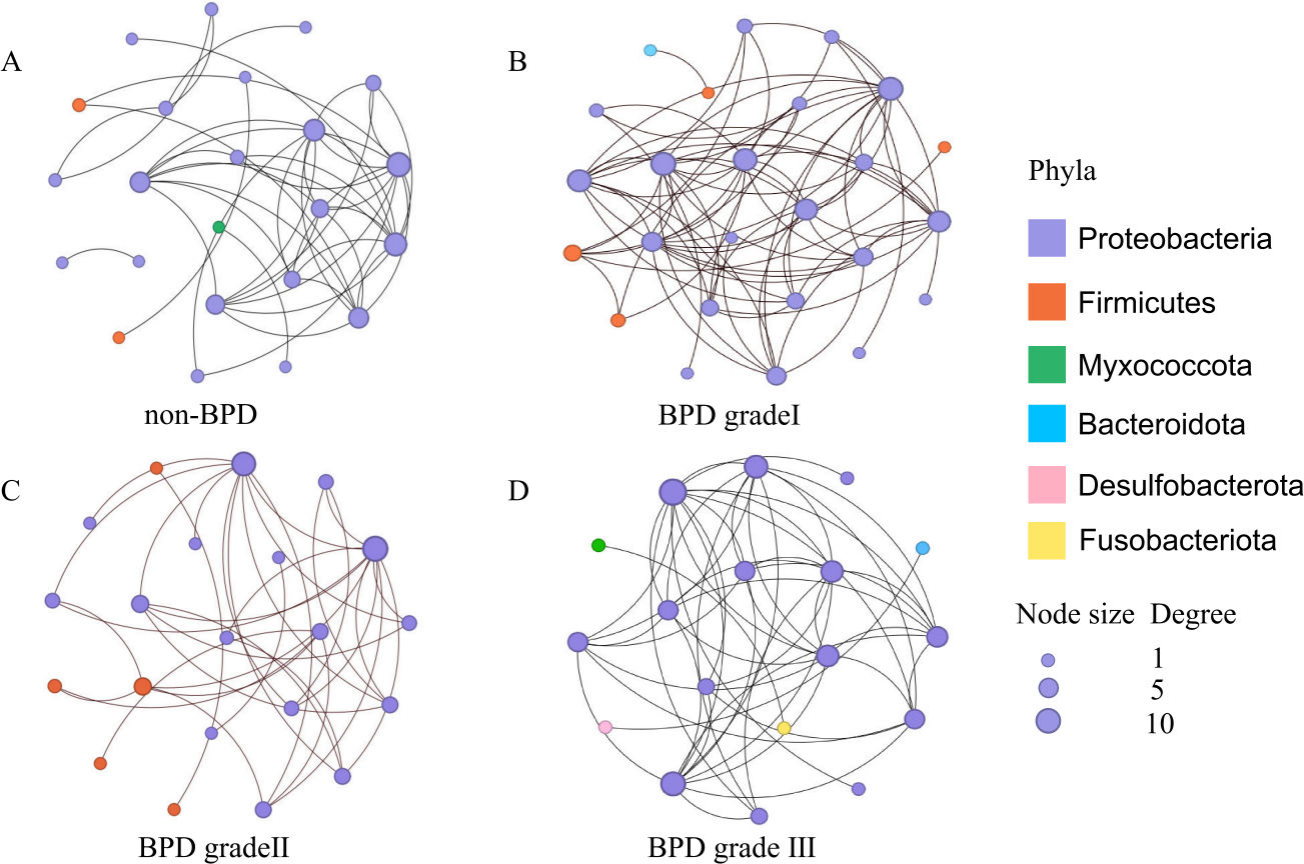
The microbial co-occurrence patterns in the lower airway microbiome at ASV levels in the non-BPD group (**A**), BPD grade I group (**B**), BPD grade II group (**C**), and BPD grade III group (**D**). The node sizes present the degree centrality. The diameter of each node is proportional to its degree centrality within its respective network.

The SparCC networks identified a conservative number of strong correlations (ranging from 35 to 72 edges across groups), whereas Spearman’s method detected a vastly larger number of associations (ranging from 2,044 to 7,989 edges). The Jaccard index, quantifying the similarity in edges identified by both methods, was minimal (0.003 to 0.023). The final, robust SparCC networks are visualized in Fig. 5. Following this, we found that the complexity of microbial co-occurrence networks dropped sharply in the BPD grade III group (Table 1). We determined that a correlation threshold of |*r*| >0.3 yielded the most robust ecological networks, as it optimally balanced the avoidance of false-positive associations with the retention of meaningful ecological information (Table S4). Using the |*r*| >0.3 threshold, we identified *Acinetobacter* and *Fusobacterium* as keystone taxa at the non-BPD group, *Brevundimonas* and *Fusobacterium* at grade I, and *Fusobacterium* and *Acinetobacter* at grade II and grade III. For the four key species, the model excluded the antibiotic type variable, which caused numerical issues and convergence failure when introduced. Therefore, all models included the remaining 10 covariates. The abundance of key species showed no significant impact on BPD levels: ASV 1303 (OR = 1.15, *P* = 0.52), ASV 708 (OR = 0.85, *P* = 0.46), ASV 1702 (OR = 0.98, *P* = 0.94), and ASV 2215 (OR = 0.89, *P* = 0.56).

**TABLE 1:** Network topological features of preterm infants of each BPD group^*a*^

Feature	Non-BPD network	BPD grade I network	BPD grade II network	BPD grade III network
Nodes	42	153	159	29
Edges	34	445	2,317	20
Average degree	1.62	5.82	29.14	1.38
Average path length	0.04	0.02	0.01	0.03
Diameter	0.01	0.12	0.09	0.08
Density	0.04	0.04	0.18	0.05
Clustering coefficient	0.23	0.01	0.72	0.23
Modularity	0.77	0.43	0.04	0.83
Betweenness centralization	0.02	0.12	0.04	0.03
Closeness centralization	0.64	1.22	0.95	0.42
Eigenvector centralization	0.05	0.13	0.38	0.04

^*a*^ BPD, bronchopulmonary dysplasia.

**Fig 6:**
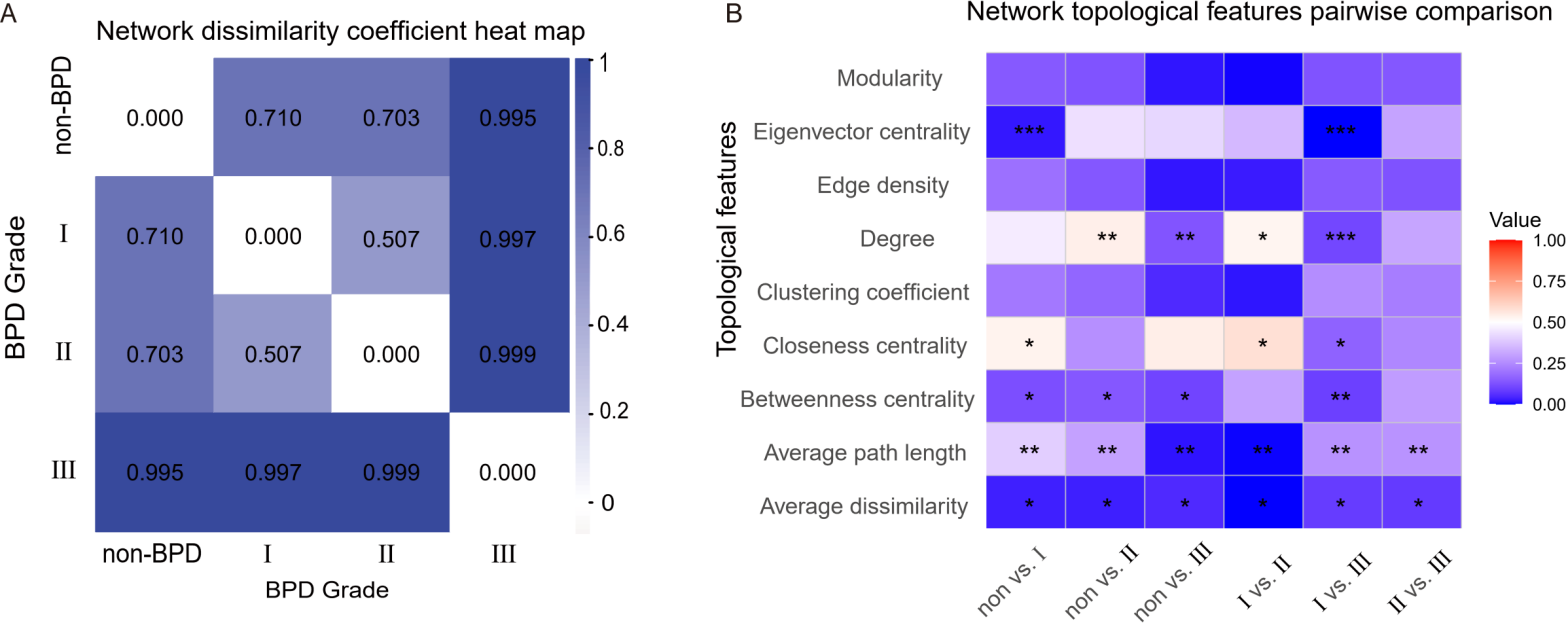
Results of network dissimilarity analysis. Network dissimilarity coefficient heat map (**A**). The color depth indicates the greater network dissimilarity coefficient. Network topology characteristic parameter heat map (**B**) shows significant differences in microbial interaction networks among groups (***: *P* < 0.001, **: *P* < 0.01, *: *P* < 0.05). The value indicates the correlation coefficient (*R*) of network topological feature between groups.

Network dissimilarity coefficients indicated that the microbial interaction network of the BPD grade III group significantly differed from others (Fig. 6A). We further compared the topological parameters among the four groups, and the results showed significant differences in average dissimilarity and average path length between any two groups. Except for the II vs III group, there were differences of centrality between the other groups. Significant differences in network degree were also found between the low-grade BPD groups and the high-grade groups (Table 2) (Fig. 6B).

**TABLE 2:** Differences between the network topology parameters compared pairwise

Parameter	Non vs I	Non vs II	Non vs III	I vs II	I vs III	II vs III
Modularity^*a*^	0.14	0.128	0.020	0.005	0.128	0.137
Eigenvector centrality^*b*^	0.022***	0.432	0.414	0.349	0.000***	0.303
Edge density^*a*^	0.191	0.137	0.021	0.027	0.144	0.127
Degree^*b*^	0.457	0.541**	0.132**	0.528*	0.103***	0.306
Clustering coefficient^*a*^	0.208	0.168	0.049	0.019	0.253	0.218
Closeness centrality^*b*^	0.533*	0.259	0.545	0.581*	0.161*	0.240
Betweenness centrality^*b*^	0.120*	0.138*	0.100*	0.300	0.091**	0.286
Average path length^*a*^	0.396**	0.298**	0.018**	0.003**	0.264**	0.267**
Average dissimilarity^*a*^	0.032*	0.031*	0.049*	0.001*	0.081*	0.079*

^*a*^ Indicates that the value is the difference between the corresponding indicators of the comparing groups.

^*b*^ Indicates that the value is the Jaccard index. Significant differences were evaluated using *P* values and indicated in the table (*: *P* < 0.05, **: *P* < 0.01; ***: *P* < 0.001).

Based on the ordinal logistic regression analysis, microbial network topology demonstrates limited independent utility in predicting BPD severity after rigorous adjustment for established clinical risk factors. After rigorous adjustment, network topology overall demonstrated limited independent predictive utility for BPD severity. However, network density emerged as a significant independent predictor, with a higher density associated with a substantially reduced risk of severe BPD (OR = 0.12, *P* < 0.05). In contrast, other topological characteristics showed no significant associations. BW emerged as a significant protective factor (OR < 1). The polynomial terms for both ACS and MSF demonstrated strong statistical significance, revealing substantial nonlinear relationships between these variables and the ordinal outcome (Table S6).

## DISCUSSION

In this prospective cohort of preterm infants, we found that the architecture of the early airway microbial co-occurrence network rather than individual taxon abundance was associated with BPD severity. The observed decline in network complexity with increasing BPD severity suggests a loss of ecological stability that may exacerbate pulmonary inflammation and disease progression.

By comparing the clinical characteristics of the participants, we found that the severity of BPD is negatively correlated with GA and BW. This aligns with previous research findings (24, 25). In our study, infants in the groups differed by the type of antibiotic therapy and mode of delivery. Infants with developing severe BPD were likely to be the least healthy, so they were treated with β-lactam antibiotics in combination with other types of antibiotics rather than β-lactam monotherapy. Cesarean section is more common in premature infants than vaginal delivery, and infants born by cesarean section are more likely to develop BPD. In terms of complications, we found that the severity of BPD is associated with retinopathy of prematurity, hsPDA, anemia, and EUGR. This may be a result of the low GA among BPD patients, and they usually have underdeveloped organs and poor nutrition. EUGR is one of the main prenatal factors that increase the incidence of BPD in preterm infants, which is consistent with our findings, and it is also associated with a higher risk of long-term respiratory disease and lung function impairment later in life (26–28).

Whether BPD occurs or not, the main microorganisms in the LRT secretions of preterm infants are Proteobacteria, Bacillota, and Bacteroidota. These results are similar to previous studies (29, 30), but there is a study that has shown that the main microorganisms in the respiratory tract of infants are *Staphylococcus* and *Ureaplasma* (31). The respiratory microbiome of neonates born vaginally is similar to that of their mothers, while those born by cesarean section carry more microbes from skin and the environment (32). In our results, no significant difference in alpha and beta diversity was found, indicating that the abundance and community structure of the airway microbiome at birth were similar.

Our findings reveal a severity-dependent restructuring of the airway microbiota in BPD. The early enrichment of *Escherichia-Shigella* and *Streptococcus* in BPD grades I–II suggests their potential role in initiating or reflecting local airway inflammation. The subsequent decline of *Streptococcus* alongside the rise of *Chryseobacterium* in the BPD grade III group may indicate a microbial shift toward a more dysbiotic airway environment, potentially associated with prolonged mechanical ventilation or clinical interventions. These patterns support the concept of stage-specific microbial signatures in BPD progression. While *Escherichia-Shigella* and *Streptococcus* could serve as early biomarkers, *Chryseobacterium* may reflect advanced disease. Future studies should investigate whether these microbial changes contribute causally to BPD pathogenesis or are secondary to disease severity and clinical management.

Our analysis identified *Acinetobacter* and *Fusobacterium* as recurrent keystone taxa across BPD severity groups. The prominence of *Acinetobacter* is particularly concerning, as its presence in the neonatal gut has been specifically linked to an increased risk of adverse outcomes in preterm infants. A longitudinal study found that *Acinetobacter* was associated with a higher risk of necrotizing enterocolitis and late-onset sepsis, suggesting its potential role in propagating systemic inflammation (33). Concurrently, *Fusobacterium* is a well-known pro-inflammatory genus. It is a potent activator of innate immunity through TLR4 signaling (34). The emergence of *Brevundimonas* in mild BPD, though less studied, hints at stage-specific microbial drivers. Together, the dynamic reorganization of these keystone taxa suggests that the airway microbiome may play a critical role in modulating inflammation and disease progression in BPD (35).

Our co-occurrence network analysis, employing the compositionally robust SparCC method, revealed that the microbial ecosystem undergoes a fundamental restructuring with increasing BPD severity. The stark contrast with conventional Spearman correlation ecosystem—which generated orders of magnitude more connections with minimal overlap—robustly underscores that the observed network simplifications are genuine ecological phenomena, not spurious artifacts of compositional data. Crucially, by modeling network metrics against clinical outcomes, we identified that this change is best characterized not by a rewiring of specific pathways, but by a systemic collapse in community connectivity. This is powerfully evidenced by our finding that a higher microbial network density was independently associated with a substantially reduced risk of severe BPD, indicating that a stable, well-connected community plays a protective role. Conversely, the microbial ecosystem in severe BPD was sparse and fragmented, suggesting a loss of ecological stability that could exacerbate pulmonary inflammation. The recurrent identification of pro-inflammatory genera like *Acinetobacter* and *Fusobacterium* as keystone taxa across severity groups provides a putative mechanism for this structural collapse. We hypothesize that these taxa act not as central hubs within a stable network, but as “hub-breakers”—their presence and activity may disrupt symbiotic interactions, outcompete beneficial taxa, and thereby erode the very fabric of the cooperative microbial community. This process would manifest as the observed global decrease in network density. Thus, the influence of key microbes appears to be ecological and systemic, driving a shift from a robust, interconnected state to a vulnerable, disconnected one. In addition, we found that there are significant differences in both average dissimilarity and average path length between any two groups. Centrality helps to assess key species in microbial communities, which play a central role in maintaining community structure and function. Differences in centrality among groups may indicate changes in key species in microbial communities under different developing states of BPD.

Several limitations should be acknowledged. The single-time-point sampling precluded longitudinal assessment of microbiome dynamics. Secondly, our 16S rRNA sequencing approach with V3–V4 primers is not optimal for detecting *Ureaplasma* and *Mycoplasma*, as confirmed by a bioinformatic assessment revealing low primer specificity. Therefore, we cannot rule out a potential role for these organisms in BPD pathogenesis within our cohort. Additionally, the modest sample size after down-sampling necessitates cautious interpretation of network comparisons.

Despite these limitations, our study strengthens the evidence linking early airway microbiome structure to BPD risk. By integrating compositionally robust network inference with clinical modeling, we provide a novel ecological perspective on BPD pathogenesis. Future studies should validate these findings in larger cohorts and explore targeted strategies to modulate microbial interactions for BPD prevention.

## Data Availability

Raw sequence data have been deposited in the sequence read archive at the NCBI (https://www.ncbi.nlm.nih.gov/) under BioProject accession number SRP584334. The metadata and full analysis code, including QIIME2 workflows, SparCC network analysis, and statistical modeling scripts, are publicly available on GitHub at https://github.com/QianZhangEMHlab/BPD_analysis_project. The repository has been archived to ensure long-term stability and is released under the MIT License.

## SUPPLEMENTAL MATERIAL

Checklist msphere.00857-25-s0001.docx STORMS checklist.

Supplemental material msphere.00857-25-s0002.docx Supplemental text, tables, and figures.

ASM does not own the copyrights to Supplemental Material that may be linked to, or accessed through, an article. The authors have granted ASM a non-exclusive, world-wide license to publish the Supplemental Material files. Please contact the corresponding author directly for reuse.
