# Supplementary material for: Co-occurrence network analysis reveals novel associations between the neonatal airway microbiome and bronchopulmonary dysplasia risk: an observational, population-based study
Source: mSphere. 2026 Feb 3;11(2):e00857-25. doi: 10.1128/msphere.00857-25 (PMC12931270; doi:10.1128/msphere.00857-25)

**Online-only Supplement**

**eMethods:** Detailed methodology

**eTable 1:** Maternal and neonatal characteristics by BPD group

**eTable 2:** Clinical treatments by BPD group

**eTable 3**: Clinical outcomes by BPD group

**eTable4**. Sensitivity analysis of keystone taxon identification across correlation thresholds

**eTable5**. Differential abundance analysis at the genus level

**eTable6**. Regression analysis for network metrics with BPD Severity

**eTable7**. BPD Severity Grading Criteria (Based on the 2018 NICHD Consensus)

**eFigure1**. CONSORT-style flow diagram

**eMethods**

**Detailed methodology**

**Extraction and Sequencing**

DNA was extracted using FastDNA® Spin Kit for Soil (MP Biomedicals) and the extracted genomic DNA was detected by agarose gel electrophoresis. Rigorous negative controls were processed in parallel with all experimental samples throughout the entire workflow. Sterile water was processed alongside samples during DNA extraction and used as template in PCR amplification. These controls were subsequently subjected to agarose gel electrophoresis under the same conditions with the actual DNA samples. Critically, no detectable DNA bands were observed in any of the negative control lanes, while the experimental samples showed clear and distinct bands. This result robustly demonstrates the absence of significant contamination in our reagents and procedures, thereby confirming the reliability of the subsequent sequencing data. (Figure2) The V3 and V4 region (338F: 5’-ACTCCTACGGGAGGCAGCAG-3’, 806R： 5’-GGACTACHVGGGTWTCTAAT-3’）of the 16S ribosomal RNA gene was sequenced on an Illumina MiSeq platform (San Diego, CA, USA). Briefly, PCR was conducted in triplicate under the following conditions: 95 °C for 3 min, followed by 30 cycles at 95 °C for 30 s, 55 °C for 30 s, and 72 °C for 45 s and a final extension at 72 °C for 10 min. Each sequencing batch included samples from all experimental groups to ensure that technical variation was balanced between groups. Sequencing libraries were prepared by using NEXTFLEX Rapid DNA-Seq Kit (Bioo Scientific). Paired-end sequencing was performed using an Illumina MiSeq PE300 platform with MiSeq Reagent Kit v3.

**Bioinformatics Processing**

Raw sequence data has been deposited in the sequence read archive at the NCBI (https://www.ncbi.nlm.nih.gov/) under BioProject accession number [SRP584334](https://trace.ncbi.nlm.nih.gov/Traces/sra?study=SRP584334). The further preprocessing of sequence data was performed on QIIME2 pipeline. Quality control and filtering of paired-end reads were conducted based on sequencing quality, and splicing was carried out according to the overlap relationship between paired-end reads to obtain optimized data after quality control splicing. Then, sequence denoising methods (DADA2) were used to process the optimized data, obtaining amplicon sequence variant (ASV) representative sequences and abundance information^1^. ASVs were clustered against the Silva reference database (ver. 138.2) and their taxonomic identities were assigned by the RDP classifier (http://rdp.cme.msu.edu/，version 2.11)^2,3,4^. Specifically, we extracted primer-specific reference sequences from the full-length 16S sequences in the Silva database. We then compared the trained classifier with the representative sequences of bacteria identified by DADA2 analysis to obtain bacterial taxonomic information. To avoid random sequencing errors and host pollution, we filtered out features that appeared only once, as well as mitochondrial and chloroplast sequences. The taxonomic annotation was subsequently updated following this filtration step.

**Network Sensitivity Analysis**

To evaluate the robustness of the microbial co-occurrence networks to methodological choices, we conducted a comprehensive sensitivity analysis.

We selected |ρ| > 0.3 as the primary threshold based on the characteristics of the SparCC method. Unlike general correlation methods (e.g., Spearman), SparCC is specifically designed for compositional data, and its estimated correlation coefficients are generally more conservative with lower absolute values. Network construction employed a threshold of |ρ| > 0.3 to define significant microbial associations. This threshold selection was based on established practices in microbial ecological network analysis^5^. An FDR < 0.05 ensures that the proportion of expected false discoveries among all edges deemed significant is below 5%, thereby strictly controlling errors statistically.

We retained only taxa that appeared in more than 5 samples and had a relative abundance greater than 0.01% to filter out extremely low-abundance or rare species that may originate from sequencing errors or environmental contamination. Removing ASVs absent in most samples helps prevent numerical instability during SparCC's log-ratio calculations, thereby yielding more reliable correlation estimates. To explore how alternative thresholds affect topology and keystone identification, we conducted sensitivity analyses using multiple thresholds with 0.2 and 0.4.

**References**

1. Callahan BJ, McMurdie PJ, Rosen MJ, Han AW, Johnson AJA, Holmes SP. DADA2: high-resolution sample inference from Illumina amplicon data. *Nat Methods*. 2016;13(7):581-583.
2. Bokulich NA, Kaehler BD, Rideout JR, et al. Optimizing taxonomic classification of marker-gene amplicon sequences with QIIME 2’s q2-feature-classifier plugin. *Microbiome*. 2018;6:90.
3. Quast C, Pruesse E, Yilmaz P, et al. The SILVA ribosomal RNA gene database project: improved data processing and web-based tools. *Nucleic Acids Res*. 2013;41(Database issue):D590-D596.
4. Wang Q . Naive Bayesian classifier for rapid assignment of rRNA sequences into the new bacterial taxonomy[J]. *Appl Environ Microbiol*, 2007, 73. doi:10.1128/AEM.00062-07.
5. Kishore D, Birzu G, Hu ZJ, DeLisi C, Korolev KS, Segre D. Inferring microbial co-occurrence networks from amplicon data: a systematic evaluation. *Msystems* 2023;8(4).

**Supplementary Tables**

**eTable 1. Maternal and neonatal characteristics by BPD group**

|  | **Non-BPD (n=31)** | **BPD grade I (n=31)** | **BPD grade II (n=20)** | **BPD grade III (n=16)** | ***p* value** |
| --- | --- | --- | --- | --- | --- |
| Sex, n (%) |  |  |  |  | .689 |
| Male | 20 (64.5) | 22 (71.0) | 11 (55.0) | 11 (68.8) |  |
| Female | 11 (35.5) | 9 (29.0) | 9 (45.0) | 5 (31.3) |  |
| Gestational Age (wk), Median (IQR) | 31.14 (2.00) | 28.86 (3.29) | 28.79 (3.68) | 27.00 (3.69) | <.001 |
| Birth weight (g),  Mean (SD) | 1500.35 (295.33) | 1241.19 (273.78) | 1173 (241.08) | 1017.81 (419.70) | <.001 |
| Maternal age (year),  Mean (SD) | 32.13 (4.99) | 30.71 (4.07) | 34.95 (5.91) | 32.38 (4.19) | .061 |
| Delivery, n (%) |  |  |  |  | .048 |
| Vaginal | 7 (22.6) | 6 (19.4) | 4 (20.0) | 9 (56.3) |  |
| Caesarean | 24 (77.4) | 25 (80.6) | 16 (80.0) | 7 (43.8) |  |
| DCC*, n (%) | 21 (67.7) | 16 (51.6) | 13 (65.0) | 8 (50.0) | .502 |
| ACS, n (%) | 29 (93.5) | 29 (93.5) | 19 (95.0) | 15 (93.7) | .926 |
| GDM, n (%) | 10 (32.3) | 11 (35.5) | 9 (45.0) | 5 (31.3) | .789 |
| HDCP, n (%) | 8 (25.8) | 2 (6.5) | 4 (20.0) | 5 (31.3) | .103 |
| ICP, n (%) | 0 (0.0) | 2 (6.5) | 0 (0.0) | 0 (0.0) | .328 |
| PROM, n (%) | 11 (35.5) | 14 (45.2) | 9 (45.0) | 5 (31.3) | .731 |
| Placental abruption, n (%) | 5 (16.1) | 8 (25.8) | 2 (10.0) | 2 (12.5) | .542 |
| Chorioamnionitis, n (%) | 3 (9.7) | 4 (12.9) | 4 (20.0) | 3 (18.8) | .678 |
| Fetal distress, n (%) | 3 (9.7) | 5 (16.1) | 3 (15.0) | 5 (31.3) | .329 |
| Thyroid disease, n (%) | 2 (6.5) | 4 (12.9) | 0 (0.0) | 1 (6.3) | .372 |
| Maternal prenatal antibiotic exposure | 18(58.1) | 23(74.2) | 9(45) | 10(62.5) | 0.531 |

Notes: *DCC, delayed umbilical cord clamping. ACS, antenatal corticosteroid. GDM, gestational diabetes mellitus. HDCP, hypertensive disorders complicating pregnancy. ICP, intrahepatic cholestasis of pregnancy. PROM, premature rupture of membrane. BPD, bronchpulmonary dysplasia.

Independent sample t-test was used for continuous variables and chi-squared test or Exact Fisher’s test was used for categorical variables.

##

## eTable2. Clinical treatments by BPD group

|  | **Non-BPD (n=31)** | **BPD grade I (n=31)** | **BPD grade II (n=20)** | **BPD grade III (n=16)** | **p value** |
| --- | --- | --- | --- | --- | --- |
| Hospital Stay (d),  Mean (SD) | 42.43 (14.53) | 64.03 (15.78) | 69.16 (19.83) | 76.44 (22.24) | <.001 |
| Anti-infective time (d), Mean (SD) | 7.19 (4.301) | 11.81 (6.84) | 12.20 (7.59) | 12.00 (8.30) | .001 |
| Antimicrobial therapy, n (%) |  |  |  |  | .008 |
| Monotherapy | 25 (80.6) | 19 (61.3) | 14 (70.0) | 5 (31.3) |  |
| Combination therapy | 6 (19.4) | 12 (38.7) | 6 (30.0) | 11 (68.8) |  |
| IMV (h),  Mean (SD) | 100.08 (76.58) | 78.85 (86.88) | 125.16 (141.60) | 98.75 (152.00) | .377 |
| NIMV (h),  Mean (SD) | 914.68 (374.59) | 770.04 (452.82) | 857.37 (464.19) | 928.25 (633.98) | .772 |
| Oxygen inhalation (h), Mean (SD) | 1229.48 (462.94) | 1090.63 (486.38) | 1207.53 (558.96) | 1312.58 (784.43) | .827 |
| PS, n (%) | 28 (90.3) | 29 (93.6) | 20 (100.0) | 12 (75.0) | .886 |
| Postnatal corticosteroids, n (%) | 17 (60.7) | 12 (41.4) | 13 (63.2) | 7 (53.8) | .401 |
| PDA treatment, n (%) | 8 (28.6) | 8 (58.6) | 11 (57.9) | 7 (53.8) | .284 |
| Blood transfusion, n (%) | 20 (74.1) | 17 (58.6) | 11 (57.9) | 7 (53.8) | .493 |

Notes: IMV, intermittent mandatory ventilation. NIMV, nasal intermittent mandatory ventilation. PS, pulmonary surfactant. PDA, patent ductus arteriosus; BPD, bronchpulmonary dysplasia

## eTable3. Clinical outcomes by BPD group

|  | **Non-BPD (n=31)** | **BPD grade I (n=31)** | **BPD grade II (n= 20)** | **BPD grade III (n=16)** | **p value** |
| --- | --- | --- | --- | --- | --- |
| IVH, n (%) | 16 (51.6)* | 14 (45.2) | 11 (55.0) | 4 (25.0) | .571 |
| PVL, n (%) | 1 (3.2) | 2 (6.5) | 2 (10.0) | 0 (0.0) | .676 |
| EOS, n (%) | 3 (9.7) | 10 (32.3) | 4 (20.0) | 4 (25.0) | .082 |
| LOS, n (%) | 2 (6.9) | 0 (0.0) | 2 (10.5) | 1 (11.1) | .216 |
| PPHN, n (%) | 9 (29.0) | 13 (41.9) | 5 (20.0) | 4 (25.0) | .573 |
| PH, n (%) | 4 (12.9) | 3 (9.7) | 6 (30.0) | 4 (25.0) | .055 |
| PALS, n (%) | 2 (6.5) | 1 (3.2) | 1 (5.0) | 0 (0.0) | .89 |
| hsPDA, n (%) | 3 (9.7) | 15 (48.4) | 8 (40.0) | 5 (55.6) | .004 |
| Hypothyroidism, n (%) | 2 (6.9) | 10 (32.3) | 3 (15.8) | 3 (31.2) | .053 |
| EUGR, n (%) | 11 (35.5) | 24 (77.4) | 16 (84.2) | 6 (37.5) | .004 |

Notes: *Data are presented as number (%). IVH, intraventricular hemorrhage. PVL, periventricular leukomalacia. EOS, early-onset sepsis. LOS, late-onset sepsis. PPHN, persistent pulmonary hypertension of the newborn. PH, pulmonary hemorrhage. PALS, pulmonary air leak syndrome. hsPDA, hemodynamically significant patent ductus arteriosus. EUGR, extrauterine growth restriction. BPD: bronchpulmonary dysplasia.

**eTable4. Sensitivity analysis of keystone taxon identification across correlation thresholds**

| **Group** | **N Keystone (0.2/0.3/0.4)*** | **Robust Multi-threshold Taxa** | **Jaccard (0.3 vs 0.4)** |
| --- | --- | --- | --- |
| Non-BPD | 18/2/1 | ASV1303, ASV1702, ASV708 | 0 |
| BPD grade I | 16/2/1 | ASV1303, ASV2215 | 0.5 |
| BPD grade II | 17/2/1 | ASV1303, ASV2215 | 0.5 |
| BPD grade III | 17/2/1 | ASV1303, ASV2215 | 0.5 |

Notes: *indicates the number of keystone identified by different threshold (0.2/0.3/0.4). Robust taxa listed were identified in at least two different thresholds. Jaccard index indicates the similarity of key species identified at thresholds of 0.3 and 0.4. BPD: bronchopulmonary dysplasia.

**eTable5. Differential abundance analysis at the genus level**

| **Genus** | **Comparison** | **lfc** | **q value** |
| --- | --- | --- | --- |
| *Escherichia-Shigella* | grade I vs. non-BPD | 2.09 | 0.004** |
| *Streptococcus* | grade I vs. non-BPD | 1.78 | 0.048* |
|  | grade II vs. non-BPD | 2.09 | 0.028* |
|  | grade I vs. III | -2.30 | 0.006** |
|  | grade II vs. III | -2.61 | 0.003** |
| *Chryseobacterium* | grade II vs. III | 1.83 | 0.011* |

Notes: This table only lists between-group comparisons that are statistically significant. * and ** indicate that q-value less than 0.05 and 0.01 respectively. lfc: log fold change; q-value: adjusted p-value; BPD: bronchpulmonary dysplasia.

**eTable6. Regression analysis for network metrics with BPD Severity**

| **Variable** | **Coefficient** | **Std_Error** | **OR** | **p value** |
| --- | --- | --- | --- | --- |
| Vertex_number | -0.76 | 1.14 | 0.47 | 0.50 |
| Edge_number | 0.89 | 0.97 | 2.42 | 0.36 |
| Density | -2.09 | 1.04 | 0.12 | 0.04 |
| Transitivity | 0.11 | 0.76 | 1.12 | 0.88 |
| Degree_centralization | 1.04 | 0.61 | 2.83 | 0.09 |
| Betweenness_centralization | -0.62 | 0.64 | 0.54 | 0.33 |
| Average_path_length | 0.32 | 0.82 | 1.37 | 0.70 |
| Gestational_age | 0.61 | 0.11 | 1.84 | <.001 |
| Birth_Weight | -0.01 | 0.00 | 0.99 | <.001 |
| Gender (Female) | -1.37 | 0.74 | 0.25 | 0.06 |
| Delivery_mode (Vaginal) | 0.26 | 0.86 | 1.30 | 0.76 |
| ACS.L | -9.07 | 0.75 | 0.00 | <.001 |
| Chorioamnionitis | 2.11 | 0.93 | 8.28 | 0.02 |
| MSF.L | 5.27 | 1.08 | 193.86 | <.001 |
| EUGR | -1.16 | 1.06 | 0.31 | 0.28 |
| Hospital_stay | 0.14 | 0.02 | 1.15 | <.001 |

Notes: This table presents the full results of the ordinal regression model examining associations between network topology metrics, clinical covariates, and BPD severity. BPD: bronchpulmonary dysplasia; OR, odds ratio; Polynomial terms (e.g., ACS.L) were included to model non-linear relationships.

**eTable7. BPD Severity Grading Criteria (Based on the 2018 NICHD Consensus)**

| **Our Study Grade** | **Corresponding NICHD (2018) Grade** | **Detailed Diagnostic Criteria** |
| --- | --- | --- |
| **No BPD** | **No BPD** | **Did not meet the diagnostic criteria for BPD** |
| **Grade I** | **Grade I** | **Assessed at 36 weeks' PMA:**  • FiO₂ = 0.21 on nCPAP/NIPPV or nasal cannula with flow ≥ 3 L/min; • FiO₂ = 0.22–0.29 on nasal cannula with flow 1~ < 3 L/min or oxyhood; • FiO₂ = 0.22–0.70 on nasal cannula with flow <1 L/min. |
| **Grade II** | **Grade II** | **Assessed at 36 weeks' PMA:**  • FiO₂ = 0.21 on invasive IPPV; • FiO₂ = 0.22–0.29 on NCPAP/NIPPV or nasal cannula with flow ≥ 3 L/min; • FiO₂ ≥ 0.30 on nasal cannula with flow 1~ < 3 L/min or oxyhood; • FiO₂ > 0.70 on nasal cannula with flow < 1 L/min. |
| **Grade III** | **Grade III** | **Assessed at 36 weeks' PMA:**  • FiO₂ > 0.21 on invasive IPPV; • FiO₂ ≥ 0.30 on nCPAP/NIPPV or nasal cannula with flow ≥ 3 L/min. |
| **Grade III A** | **Grade III A** | **Between 14 days postnatal age and 36 weeks' PMA:**  • Death attributable to persistent parenchymal lung disease and respiratory failure, not attributable to other neonatal comorbidities. |

Notes: BPD: bronchpulmonary dysplasia; FiO₂: **fraction of inspired oxygen; nCPAP: n**asal **c**ontinuous **p**ositive **a**irway **p**ressure; IPPV: **i**nvasive **p**ositive **p**ressure **v**entilation; NIPPV:**n**on-**i**nvasive **p**ositive **p**ressure **v**entilation; NICHD: **Eunice Kennedy Shriver National Institute of Child Health and Human Development;** PMA: p**ostmenstrual age.**

**eFigure1. Participant Flow Diagram for Microbiome Network Analysis.**

Groups were randomly down-sampled to N=16 for network construction to match the sample size.


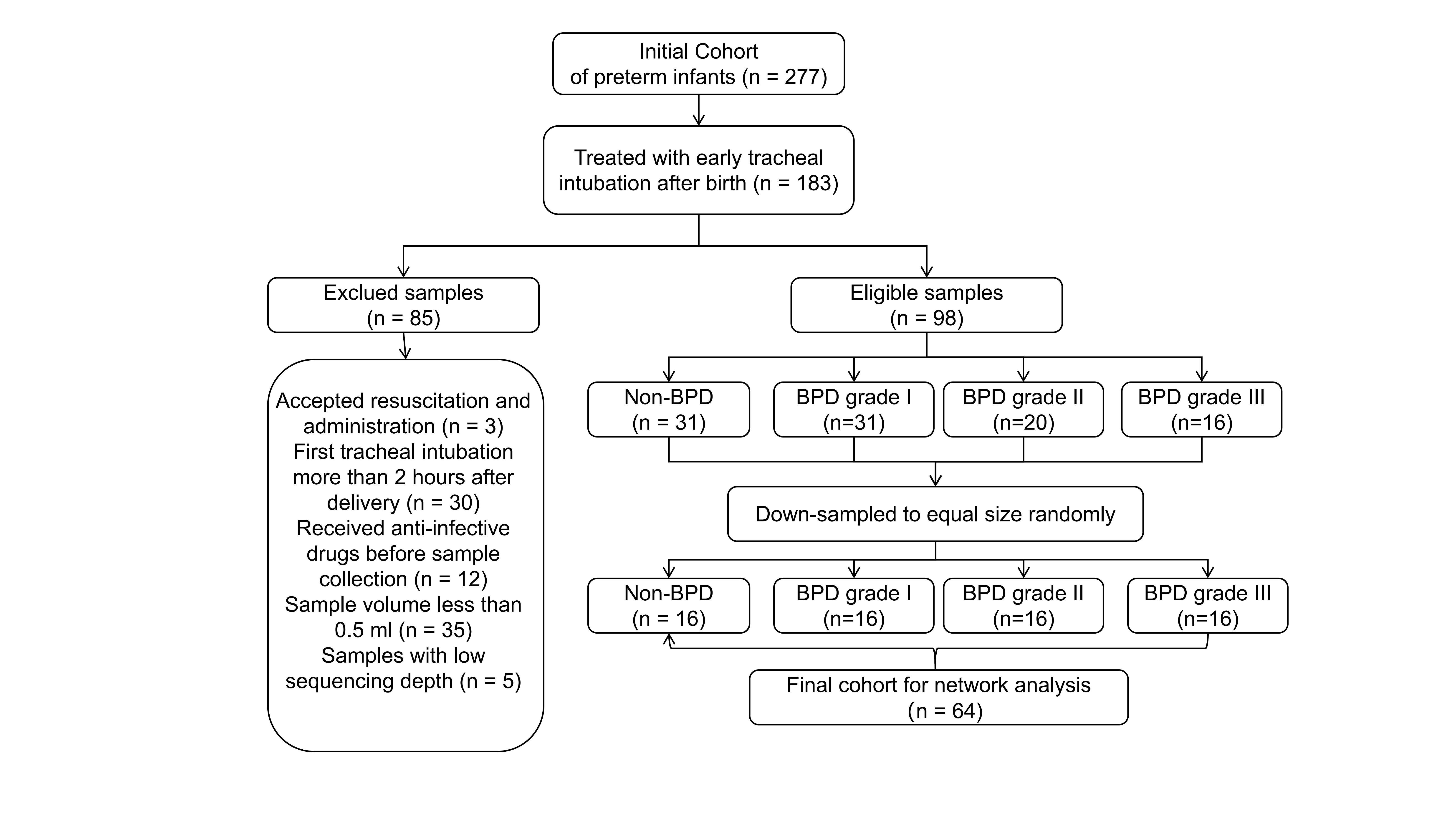

Supplement: Supplemental material — Supplemental text, tables, and figures. [file msphere.00857-25-s0002.docx]
